# Diagnostic Value of Preoperative uPAR-PET/CT in Regional Lymph Node Staging of Oral and Oropharyngeal Squamous Cell Carcinoma: A Prospective Phase II Trial

**DOI:** 10.3390/diagnostics13213303

**Published:** 2023-10-25

**Authors:** Mads Lawaetz, Anders Christensen, Karina Juhl, Giedrius Lelkaitis, Kirstine Karnov, Esben Andreas Carlsen, Birgitte W. Charabi, Annika Loft, Dorota Czyzewska, Christian von Buchwald, Andreas Kjaer

**Affiliations:** 1 Department of Otolaryngology, Head and Neck Surgery and Audiology, Rigshospitalet, Copenhagen University Hospital, 2100 Copenhagen, Denmark; mads.lawaetz@regionh.dk (M.L.);; 2 Department of Clinical Physiology, Nuclear Medicine and PET and Cluster for Molecular Imaging, Copenhagen University Hospital—Rigshospitalet & Department of Biomedical Sciences, University of Copenhagen, 2100 Copenhagen, Denmarkdorota.czyzewska@regionh.dk (D.C.); 3 Department of Pathology, Rigshospitalet, Copenhagen University Hospital, 2100 Copenhagen, Denmark

**Keywords:** urokinase-type plasminogen activator receptor (uPAR), PET/CT, ^68^Ga-NOTA-AE105, lymph node metastases, head and neck cancer

## Abstract

The detection of lymph node metastases is a major challenge in oral and oropharyngeal squamous cell carcinoma (OSCC and OPSCC). ^68^Ga-NOTA-AE105 is a novel positron emission tomography (PET) radioligand with high affinity to urokinase-type plasminogen activator receptor (uPAR), a receptor expressed on the surfaces of tumor cells. The aim of this study was to investigate the diagnostic value of uPAR-PET/CT (computerized tomography) in detecting regional metastatic disease in patients with OSCC and OPSCC compared to the current imaging work-up. In this phase II trial, patients with OSCC and OPSCC referred for surgical treatment were prospectively enrolled. Before surgery, ^68^Ga-NOTA-AE105 uPAR-PET/CT was conducted, and SUVmax values were obtained from the primary tumor and the suspected lymph nodes. Histology results from lymph nodes were used as the standard of truth of metastatic disease. The diagnostic values of ^68^Ga-uPAR-PET/CT were compared to conventional routine preoperative imaging results (CT and/or MRI). The uPAR expression in resected primary tumors and metastases was determined by immunohistochemistry and quantified digitally (H-score). A total of 61 patients underwent uPAR-PET/CT. Of the 25 patients with histologically verified lymph node metastases, uPAR-PET/CT correctly identified regional metastatic disease in 14 patients, with a median lymph node metastasis size of 14 mm (range 3–27 mm). A significant correlation was found between SUVmax and the product of the H-score and tumor depth (r = 0.67; *p* = 0.003). The sensitivity and specificity of uPAR-PET/CT in detecting regional metastatic disease were 56% and 100%, respectively. When added to CT/MRI, uPAR-PET was able to upstage 2/11 (18%) of patients with occult metastases and increase the sensitivity to 64%. The sensitivity and specificity of ^68^Ga-NOTA-AE105 uPAR-PET/CT were equivalent to those of CT/MRI. The significant correlation between SUVmax and uPAR expression verified the target specificity of ^68^Ga-NOTA-AE105. Despite the target specificity, the sensitivity of imaging is too low for nodal staging and it cannot replace neck dissection.

## 1. Introduction

Oral (OSCC) and oropharyngeal (OPSCC) squamous cell carcinomas are two of the most frequent malignancies of the head and neck [1]. The occurrence of cervical lymph node metastases is the most important clinical prognostic factor [2,3,4,5]. The detection of lymph node metastases remains a major challenge in OSCC and OPSCC, where 21–33% of patients without clinically suspicious regional lymph nodes (cN0 neck) have occult regional metastases at the time of diagnosis that are not detected on magnetic resonance imaging (MRI), computed tomography (CT) or ultrasound [6,7,8]. Other imaging modalities, like ^18^F-FDG-PET, play an important role in head and neck squamous cell carcinoma (HNSCC), particularly in post-treatment evaluation, but lack the sensitivity to replace sentinel node biopsy or elective neck dissection, for staging and surgical planning [9]. Sentinel node biopsy is a minimally invasive method of staging the clinically and radiologically cN0 neck in patients with early-stage OSCC or OPSCC, and it has demonstrated equivalency with elective neck dissection [10]. The accurate and noninvasive identification of patients with lymph node metastases is critical in selecting the most effective treatment and reducing the frequency of unnecessary neck procedures. Unfortunately, there are currently no noninvasive imaging methods that can accomplish this.

Urokinase-type plasminogen activator receptor (uPAR) is a glyciphosphatidyliunositol (GPI)-anchored cell membrane receptor that facilitates cell invasion and metastasis by converting plasminogen into plasmin at the cell surface and thus degrading the extracellular matrix [11]. uPAR has been found upregulated in tumor cells in many solid cancers, including HNSCC, with low or absent expression in normal tissue [12,13,14,15]. Due to the tumor-specific expression of uPAR and its significance in cancer, our research group developed ^68^Ga-NOTA-AE105, a novel PET radioligand with a strong affinity to uPAR. The biodistribution, safety and tumor detection ability of ^68^Ga-NOTA-AE105 have been investigated in a phase I study [16], and recent phase II studies have shown the significant prognostic value of uPAR-PET/CT in patients with HNSCC [17] and in patients with neuroendocrine neoplasms [18]. However, the diagnostic value of uPAR-PET/CT in OSCC and OPSCC has not yet been explored.

The aim of this phase II study was therefore to investigate the diagnostic value of ^68^Ga-NOTA-AE105 uPAR-PET/CT in detecting regional metastatic disease in patients with OSCC and OPSCC compared to the current imaging work-up.

## 2. Materials and Methods

### 2.1. Study Design and Patients

**Figure 1:**
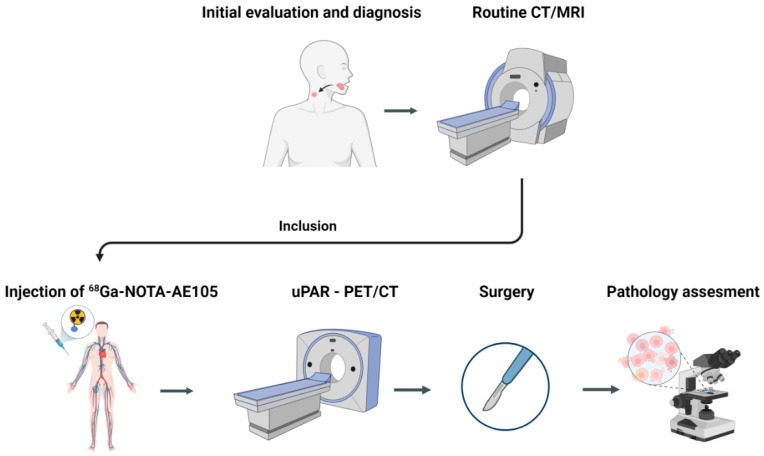
The study design. Patients, regardless of lymph node status, were included after routine evaluation and imaging. Following uPAR-PET/CT, patients underwent surgical excision of the primary tumor and the removal of regional lymph nodes (sentinel node dissection and/or elective neck dissection). uPAR-PET/CT-positive lymph nodes were finally compared to the pathology results.

In this prospective phase II trial, patients with biopsy-verified OSCC and OPSCC, with or without suspicion of regional neck metastases, referred for primary surgery at the Department of Otolaryngology, Head & Neck Surgery and Audiology, Rigshospitalet, Copenhagen University Hospital, Denmark, were prospectively enrolled between November 2016 and January 2022. Patients between the ages of 18 and 85 years, who were able to read, comprehend and provide informed consent, were eligible. Exclusion criteria were previous surgery or radiation therapy to the neck, obesity (bodyweight > 140 kg), allergy to ^68^Ga-NOTA-AE105 or pregnancy. Patients were enrolled in this trial following a standard evaluation and imaging work-up. The study design is shown in Figure 1. From patient records, data on age, gender, stage, preoperative imaging modalities, laboratory findings and histology results were obtained. Patients’ disease stage was classified according to the 8th edition of the Union for International Cancer Control (UICC) staging manual [19]. Patients included between 2016 and 2018, i.e., prior to the introduction of the 8th edition of the UICC staging manual, were subsequently reclassified according to this edition, so that all patients were classified according to the most recent TNM staging manual. The study was approved by the Danish Research Ethics Committee (protocol no. H-16032922) and the Danish Medicines Agency (protocol no. 2016122500). The trial was registered in the European Union Drug Regulating Authorities Clinical Trials Database (EudraCT no. 2016-002360-14) and on ClinicalTrials.gov (Identifier: NCT02960724) and conducted in compliance with the Good Clinical Practice (GCP) recommendations.

### 2.2. Image Acquisition

The radioligand, ^68^Ga-NOTA-AE105, was produced in-house as previously published [16]. Before surgery, patients underwent a whole-body PET/CT scan 20 min after the injection of approximately 200 MBq (median 199 MBq, range 112–214 MBq) of ^68^Ga-NOTA-AE105. Whole-body PET and diagnostic CT with iodine intravenous contrast (skull base to proximal thigh) were performed in the same session with an integrated whole-body PET/CT system (Siemens Biograph mCT 64 slice, Siemens, Munich, Germany) with patients placed in a supine position. The PET data were reconstructed using an iterative reconstruction technique that used time of flight, point spread function and attenuation correction with 2 iterations, 21 subsets and a 2 mm Gaussian filter. The CT scan was carried out with 120 kV, 170 mAs and a pitch of 0.8. Any adverse events were recorded within 24 h following ^68^Ga-NOTA-AE105 injection.

### 2.3. Image Analysis

All ^68^Ga-uPAR-PET/CT scans were evaluated by an experienced physician in nuclear medicine and an experienced radiologist working side by side, both blinded to all clinical data, including the TNM stage and results from the previous routine imaging work-up (CT/MRI). The lymph nodes were classified as positive if the team visually found higher uptake in a lymph node compared with surrounding normal tissue. In the case of a positive lymph node on uPAR-PET/CT, the volume of the entire lymph node was contoured and, from this, the maximum standardized uptake value (SUV_max_) was obtained. The radiologist determined the anatomic lymph node level of each uPAR-PET/CT-positive lymph node. Later, the neck uPAR-PET/CT results were compared to the histology report (considered the gold standard) in which the dissected levels were documented. The diagnostic performance of uPAR-PET/CT was determined as the presence or absence of lymph node metastases in a neck region compared to the histology report. The results from uPAR-PET/CT were compared with results from the previous routine imaging work-up (CT/MRI).

### 2.4. Tissue Selection and Immunohistochemistry

A specialized head and neck pathologist analyzed all resected primary tumor specimens and lymph nodes. The maximum depth of the primary tumor was measured microscopically. All resected tissue was formalin-fixated and paraffin-embedded. Smaller nodes were embedded in paraffin in toto, whereas larger nodes were divided and then embedded. Representative diagnostic slides were obtained from alle blocks as part of the routine pathology examination.

All formalin-fixated paraffin-embedded tumor samples from resected primary tumors and lymph node metastases were collected for this study. uPAR expression was determined by immunohistochemistry on 4 μm slides. Slides were incubated in 60 °C for 60 min and afterwards deparaffinized with xylene and rehydrated in decreasing grades of alcohol. Antigen retrieval was carried out in CC1 antigen retrieval buffer (Ventana Medical Systems, Tucson, AZ, USA) for 10 min at 95 °C. Endogenous peroxidase was blocked for 8 min with peroxidase-blocking solution (DAKO s2023). Blocking for unspecific antibody binding was performed using 2% BSA. Slides were incubated with uPAR-specific antibody (GeneTex, Irwine, CA, USA, product no. GTX100467, concentration 1:500) for 1 h. After this, the slides were incubated with the secondary antibody (DAKO anti-rabbit K4003) for 45 min. Staining was visualized using the DAB+ substrate chromogen system (DAKO K3468) and the specimens were lastly stained with hematoxylin for 60 s. The primary antibody was used at optimal dilution using positive and negative control staining.

### 2.5. Immunohistochemistry Scoring

An experienced head and neck pathologist digitally annotated the tumor compartments in both primary tumors and lymph node metastases and excluded necrotic areas using the open-source software Qupath version 0.3.2 [20]. Within the tumor compartment, cells were digitally identified as positive or negative based on the mean DAB signal in the cell cytoplasm. Cell expansion was set to 5 μm and the intensity threshold was set to 0.12 for weak intensity (+1), 0.25 for moderate intensity (+2) and 0.50 for strong intensity (+3). From these settings, the positive proportion of tumor cells and the H-score within the tumor compartment were calculated. The H-score ranged from 0 to 300 based on the following formula: 3 × percentage of strongly stained cells + 2 × percentage of moderately stained cells + percentage of poorly stained cells. The product of the H-score and tumor depth were correlated to SUV_max_ for all primary tumors.

### 2.6. Statistical Analysis

Sensitivity, specificity and negative and positive predictive values (NPV and PPV) were calculated per patient. McNemar’s test was used for comparison between uPAR-PET/CT and standard-of-care tests (CT/MRI). Pathology results from surgery were used as the gold standard. Correlation between SUV_max_ and the histology findings in primary tumors was performed using Spearman’s rank correlation test. The Mann–Whitney U test was used to compare the mean tumor depth between groups. All continuous values are reported as the median and range or mean ± SD. A *p*-value less than 0.05 was regarded as statistically significant. All statistical analyses were performed using IBM SPSS statistics version 25.0.

## 3. Results

### 3.1. Patients

**Figure 2:**
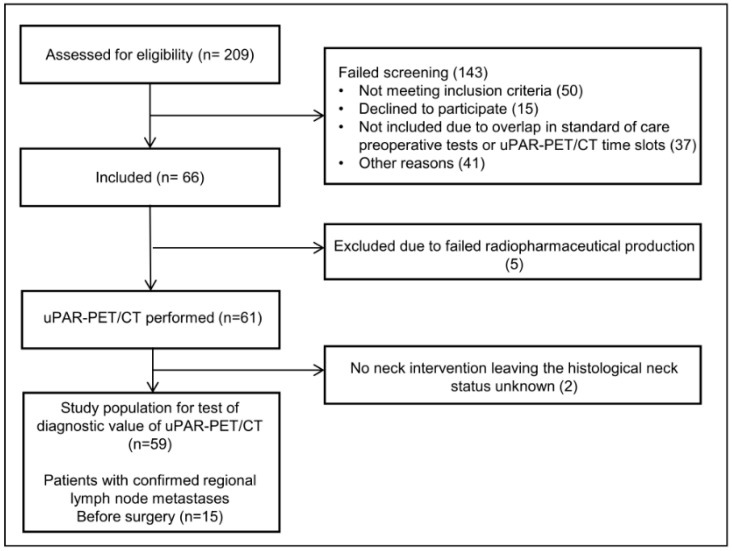
CONSORT flow diagram of the study.

We included 66 patients with OSCC and OPSCC in this phase II trial between November 2016 and January 2022. Five patients were excluded due to failed radiopharmaceutical production, leaving 61 patients with an ^68^Ga-NOTA-AE105uPAR-PET/CT for the final analyses (Figure 2). No adverse reactions or clinically detectable side effects related to the radioligand administration were observed. Patient characteristics are shown in Table 1. The median age of patients was 66 years, and the majority (79%) were diagnosed with OSCC. Sixty percent of patients were diagnosed in the early stage (stage I-II) and the vast majority (79%) of patients had small (T1-T2) primary tumors. Of the 13 patients with oropharyngeal cancer, ten patients had p16-positive tumors, eight of them with confirmed HPV-positive status (two not tested). All patients underwent standard-of-care examinations (CT and/or MRI), and 15/61 (23%) patients were preoperatively diagnosed with image- or pathology-verified lymph node metastases. The neck was managed with sentinel node biopsy (39%), selective neck dissection (51%) or a combination of both (7%). Sentinel node biopsies were only performed for patients with preoperative N0 neck. One of the 24 patients who underwent sentinel node biopsy was diagnosed with a lymph node metastasis. This patient was subsequently treated with a neck dissection, but no additional lymph node metastases were found. Two patients elected observation over neck surgery, leaving the N-stage histologically unconfirmed. Ten patients were upstaged to pN+ due to the detection of subclinical lymph node metastases following surgery, bringing the total number of patients with histologically verified regional metastases to 25 (41%). The median time from uPAR-PET/CT to surgery was 2 days (range 1–12).

### 3.2. Diagnostic Value of uPAR-PET/CT

**Figure 3:**
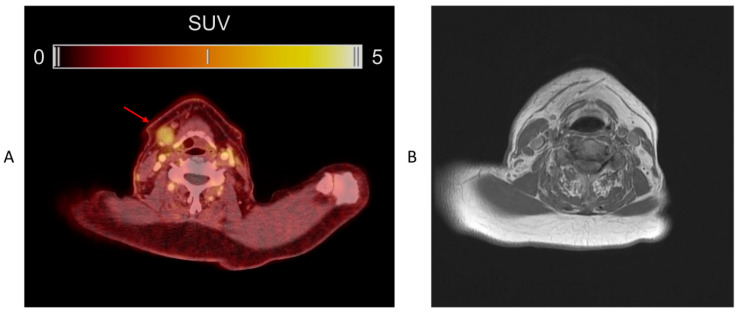
(**A**) ^68^Ga-NOTA-AE105 uPAR-PET/CT of a patient with lymph node metastasis, not detected by routine imaging work-up (CT/MRI). (**B**) MRI from the same patient.

In total, 59 patients with a neck intervention and histology-defined neck status were included in the calculation of sensitivity, specificity, PPV and NPV (Table 2). Among the 25 patients with histologically verified lymph node metastases, uPAR-PET/CT found regional metastatic disease in 14 patients (example shown in Figure 3). The same number of patients were also identified with regional metastatic disease after standard-of-care preoperative CT/MRI. However, there was a discordance between CT/MRI and uPAR-PET/CT in four cases. In two patients, uPAR-PET/CT correctly detected lymph node disease while CT/MRI was negative, whereas the inverse was observed in the two other patients. The lymph node metastases not identified by uPAR-PET/CT were significantly smaller than those detected (*p* = 0.006), with a median size in the undetected of 5 mm (range 0.1–10), compared to the median size in the detected of 14 mm (range 3–27 mm). For both uPAR-PET/CT and CT/MRI, the sensitivity and specificity on a per patient basis were 56% and 100%, respectively (no significant differences).

If standard-of-care examinations were combined with uPAR-PET/CT, the sensitivity could be increased from 56% to 64% and the NPV from 76% to 79%. An additional 2 of 11 (18%) patients with occult metastases would have been upstaged. uPAR-PET/CT was not able to detect additional metastases in patients with clinically N+ neck (patients with cervical lymph nodes already detected by routine clinical/imaging work-up).

The median SUV_max_ for lymph node metastasis was 2.62 (range 1.81–4.57); for primary tumors, it was 2.82 (range 2.00–4.40). Due to the presence of several small and superficial primary tumors, the image analysis team was only able to measure SUV values for 18/61 primary tumors for the comparison of SUV values and uPAR expression. The primary tumors identified by CT were significantly larger than the undetected tumors, with a mean tumor depth of 10.2 ± 6.2 mm versus 4.6 ± 3.5 mm (*p* < 0.001), respectively.

### 3.3. Immunohistochemistry

**Figure 4:**
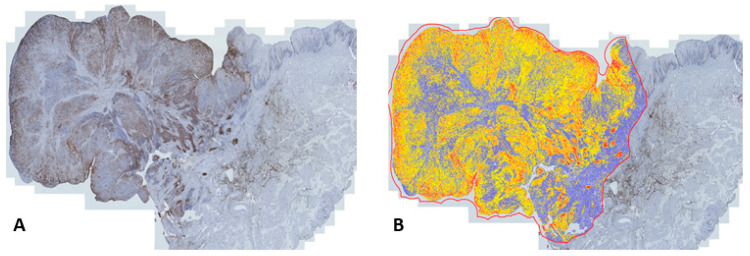
(**A**) uPAR expression in the primary tumor compartment determined with immunohistochemistry. (**B**) Digital scoring of staining intensity using Qupath (yellow = 1+, orange = 2+ and red = 3+).

**Figure 5:**
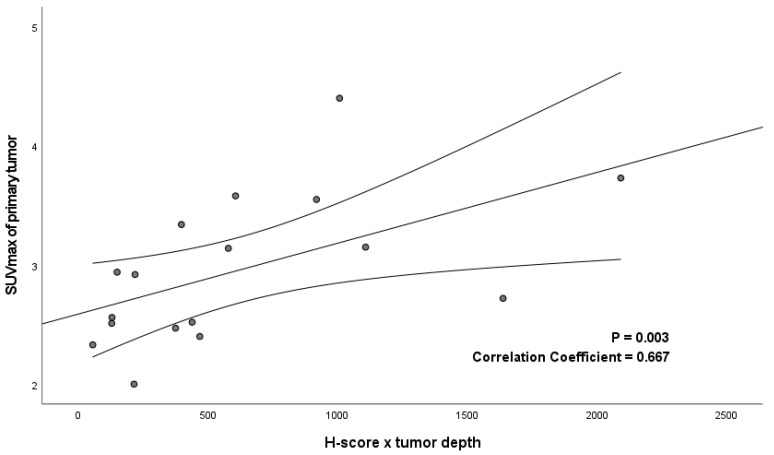
Correlation between SUV_max_ and the immunohistochemical uPAR expression expressed as the product of H-score and tumor depth in 17 primary tumors.

All primary tumors and lymph node metastases detected by CT exhibited uPAR-expression. Seventeen of the eighteen primary tumors with measurable SUV values were available for immunohistochemical examination and were assessed digitally using Qupath (Figure 4). The mean proportion of positive cells in the tumor compartments of the uPAR-PET/CT-detected tumors was 44.7 ± 22.7% and they had an H-score of 66.9 ± 36.2. A significant correlation was found in primary tumors between the SUV_max_ and the product of the H-score and tumor depth (*p* = 0.003; r = 0.67) (Figure 5). There was not sufficient tissue available from lymph node metastases to obtain a meaningful correlation between the SUV values and immunohistochemistry results.

## 4. Discussion

This prospective phase II trial including 61 patients is the first study to examine the diagnostic value of uPAR-PET/CT in patients surgically treated for OSCC and OPSCC. We found that ^68^Ga-NOTA-AE105 uPAR-PET/CT had, on a per-patient basis, sensitivity and specificity in detecting regional metastatic disease of 56% and 100%. All patients with a uPAR-PET/CT-positive neck had a histologically verified nodal malignancy, which resulted in a positive predictive value of 100%. Additionally, we found a significant correlation between SUV_max_ in the primary tumor tissue and uPAR expression, demonstrating the uPAR specificity of the PET signal.

uPAR-PET/CT and CT/MRI detected an equal number of individuals with lymph node disease; however, a discordance was seen in four patients. As a result, the combination of these modalities enhanced the diagnostic value and enabled the detection of 2/11 (18%) patients with occult metastases, rendering the sentinel node procedure unnecessary for this group. These findings suggest that uPAR-PET in combination with CT/MRI could be used to enhance the diagnostic value for tumor tissue detection in OSCC and OPSCC.

In this study, we used the in-house-produced radiotracer composed of the uPAR-specific peptide AE105, the chelator NOTA and the radiometal ^68^Ga. The advantage of ^68^Ga-labeled peptides is that they can be produced without the need for a cyclotron onsite [21]. However, the short half-life of ^68^Ga (T½ = 68 min) also represents challenges as it needs to be radiolabeled onsite, requiring radiochemistry laboratories. Using ^64^Cu (T_1/2_: 12.7 h) instead would allow for the central production and distribution of the radiopharmaceutical. Furthermore, the positron range for ^68^Ga (4 mm) is longer than that of, e.g., ^64^Cu (1 mm), which could increase the detection of smaller tumor deposits due to the increased spatial resolution, as demonstrated by our group in a head-to-head comparison of ^64^Cu and ^68^Ga-based PET tracers in neuroendocrine tumors [22]. This issue was also reported in a study using a ^68^Ga-labeled radiotracer for the PET/CT imaging of lymph node metastases in prostate cancer, which concluded that the detection rate was influenced by the metastasis size [23]. Prior animal and phase I studies have shown good resolutions with the ^64^Cu-based uPAR-PET tracer ^64^Cu-DOTA-AE105. Accordingly, it could be interesting to explore this uPAR-PET tracer in OSCC and OPSCC patients in future studies to increase the detection of occult metastasis and smaller lesions [24,25]. In our study, indeed, we experienced a challenge in identifying smaller quantities of tumor tissue, as both primary tumors and metastases not detected by ^68^Ga uPAR-PET/CT were significantly smaller than those detected. Nonetheless, some primary tumors and metastases of reasonable size were not detected by ^68^Ga-uPAR-PET/CT, indicating that the magnitude of the PET signal is not dependent on the tumor size alone, but may be a combination of the uPAR expression in tumor cells and the tumor volume. This was supported by the significant correlation between SUV_max_ and the product of the H-score and tumor depth (Figure 5). Furthermore, this correlation confirms findings from the previous phase I study that the uPAR-PET-signal in tumors is uPAR-specific [24,26].

So far, the use of PET in HNSCC has focused on ^18^F-fluorodeoxyglucose (^18^F-FDG) [27]. It has been shown that the combination of ^18^F-FDG-PET and CT is useful in detecting unknown primary tumors, secondary primary tumors and distant metastases [28,29], but the detection of small metastases in patients with a clinically N0 neck has been a challenge [30]. However, a recent prospective multicenter study investigating the diagnostic value of ^18^F-FDG-PET/CT in patients with HNSCC showed a high negative predictive value for N0 neck in patients with more advanced disease, i.e., T2–T4 tumors [31]. In recent years, there has been increasing interest in a new PET tracer, ^68^Ga-labeled fibroblast activation protein inhibitor (^68^Ga-FAPI), in different cancers, including head and neck. Fibroblast activation protein (FAP) has, in immunohistochemical studies, been found upregulated in various head and neck malignancies [32,33]. Imaging with ^68^Ga-FAPI PET/CT in head and neck cancer has demonstrated high-contrast imaging in both primary tumors and metastases and low uptake in healthy tissue [34]. For the staging of HNSCC, ^68^Ga-FAPI PET/CT has also demonstrated promising results in prospective trials, and it was recently found to outperform ^18^FDG-PET/CT in preoperative lymph node staging [35].

It was not possible to compare uPAR-PET/CT to ^18^F-FDG-PET/CT in our study due to the short window of time between diagnosis and surgery and because ^18^F-FDG-PET is not a part of the normal imaging work-up prior to surgery at our institution. However, neither ^18^F-FDG-PET/CT nor uPAR-PET/CT appear capable of replacing sentinel node biopsies or elective neck dissection in the nodal staging of OPSCC and OSCC. However, uPAR-PET/CT might have another role in patients with OSCC and OPSCC; a recent study investigating the prognostic value of ^68^Ga-NOTA-AE105 uPAR-PET/CT in HNSCC patients referred for curatively intended radiotherapy revealed that the SUV_max_ value is a prognostic factor for recurrence and may be a tool to identify patients with a high risk of recurrence [17]. Furthermore, uPAR-PET/CT has the potential to serve as a diagnostic tool to select patients for uPAR-targeted optically guided surgery, a modality currently being tested in a phase II trial in OSCC and OPSCC patients (EudraCT no. 2022-001361-12), or uPAR-targeted radionuclide therapy, which has previously been demonstrated to be effective in animal models of human prostate cancer [36] and colorectal cancer [37].

This study had some limitations. First, the CT/MRI was performed and described as part of the clinical routine examination; thus, the radiologist was not blinded to the clinical information gathered prior to imaging (e.g., clinical description, ultrasound or fine needle aspiration). In contrast, the uPAR PET/CT operator was blinded to all clinical data; consequently, there was a risk of uPAR PET/CT underperformance in comparison to CT/MRI. Secondly, this was a relatively small study and some of the findings may need to be substantiated in larger phase III studies.

## 5. Conclusions

This phase II study, evaluating the diagnostic value of ^68^Ga-NOTA-AE105 uPAR-PET/CT in identifying lymph node metastases in patients with OSCC or OPSCC, showed sensitivity and specificity equivalent to CT/MRI, with limitations in identifying smaller volumes of tumor tissue. Adding uPAR-PET to the current imaging work-up led to the identification of an additional 18% of patients with occult metastatic disease. In addition, a strong correlation was found between the uPAR expression in primary tumors and the ^68^Ga-NOTA-AE105 uPAR-PET signal measured as SUV_max_, confirming the uPAR specificity of the radiotracer. However, despite the target specificity and the ability to increase the sensitivity when added to CT/MRI, the sensitivity is too low for nodal staging and this method cannot replace neck dissection.

## Figures and Tables

**Table 1:** Characteristics of included patients and their tumor stages. All included patients were treated with primary surgery at the T-site. Neck interventions were performed as part of the primary surgery.

Characteristics	Value
**Age (years)**	Median, 66; range, 39–80
**Male/female (n)**	21/40 (34%/66%)
**Primary site (n)**	
Oral cavity	48 (79%)
Oropharynx	13 (21%)
**Treatment on N-site**	
Sentinel node	24 (39%)
Oropharynx (N0/N+)	0/0
Oral (N0/N+)	23/1
Selective neck	31 (51%)
Combination	4 (7%)
No neck surgery	2 (3%)
**Stage (n)**	
pI	34 (55%)
pII	6 (10%)
pIII	7 (11.5%)
pIV	14 (23%)
**T-stage (n)**	
pT1	31 (51%)
pT2	17 (28%)
pT3	2 (3%)
pT4	11 (18%)
**Preoperative N-stage (n)**	
cN0	46 (75%)
cN+	15 (25%)
**Postoperative N-stage (n)**	
pN0	36 (59%)
pN+	25 (51%)

**Table 2:** Diagnostic accuracy of uPAR PET/CT compared to CT/MRI for assessment of regional metastatic disease in patients with OSCC and OPSCC using pathology results as gold standard. uPAR: urokinase-type plasminogen activator receptor. pN+: pathology-verified lymph node metastatic disease. pN0: no lymph node metastases after pathology assessment of surgically removed lymph nodes.

		Pathology Results, n (%)		
Imaging Modality		Positive (pN+)	Negative (pN0)	Total
**uPAR-PET/CT**	Positive	14	0	14
	Negative	11	34	45
	Total	25	34	59
	**Sensitivity = 56%**	**Specificity = 100%**	**PPV = 100%**	**NPV = 76%**
**CT/MRI** **(standard examinations)**	Positive	14	0	14
	Negative	11	34	45
	Total	25	34	59
	**Sensitivity = 56%**	**Specificity = 100%**	**PPV = 100%**	**NPV = 76%**
**uPAR-PET/CT and CT/MRI** **combined**	Positive	16	0	16
	Negative	9	34	43
	Total	25	34	59
	**Sensitivity = 64%**	**Specificity = 100%**	**PPV = 100%**	**NPV = 79%**

## Data Availability

Data are not publicly available due to protection of personal data and medical confidentiality.
